# Multi-level exploration of auricular acupuncture: from traditional Chinese medicine theory to modern medical application

**DOI:** 10.3389/fnins.2024.1426618

**Published:** 2024-09-23

**Authors:** Kaixin Guo, Yan Lu, Xiuping Wang, Yunfeng Duan, Hui Li, Fengxiao Gao, Jian Wang

**Affiliations:** ^1^Department of Acupuncture, Shandong University of Traditional Chinese Medicine, Jinan, China; ^2^Department of Acupuncture and Moxibustion, The First Affiliated Hospital of Shandong University of Traditional Chinese Medicine, Jinan, China

**Keywords:** auricular acupuncture, nerve stimulation, pain management, metabolic disorders, psychological wellbeing, immune regulation

## Abstract

As medical research advances and technology rapidly develops, auricular acupuncture has emerged as a point of growing interest. This paper delves into the intricate anatomy of auricular points, their significance and therapeutic principles in traditional Chinese medicine (TCM), and the underlying mechanisms of auricular acupuncture in contemporary medicine. The aim is to delve deeply into this ancient and mysterious medical tradition, unveiling its multi-layered mysteries in the field of neurostimulation. The anatomical structure of auricular points is complex and delicate, and their unique neurovascular network grants them a special status in neurostimulation therapy. Through exploration of these anatomical features, we not only comprehend the position of auricular points in TCM theory but also provide a profound foundation for their modern medical applications. Through systematic review, we synthesize insights from traditional Chinese medical theory for modern medical research. Building upon anatomical and classical theoretical foundations, we focus on the mechanisms of auricular acupuncture as a unique neurostimulation therapy. This field encompasses neuroregulation, pain management, psychological wellbeing, metabolic disorders, and immune modulation. The latest clinical research not only confirms the efficacy of auricular stimulation in alleviating pain symptoms and modulating metabolic diseases at the endocrine level but also underscores its potential role in regulating patients’ psychological wellbeing. This article aims to promote a comprehensive understanding of auricular acupuncture by demonstrating its diverse applications and providing substantial evidence to support its broader adoption in clinical practice.

## 1 Introduction

TCM boasts a rich history, encapsulating centuries of medical exploration, wisdom, and experience. Auricular acupuncture, as a unique treatment modality in TCM, has been extensively documented in ancient medical classics such as the Yellow Emperor’s Inner Canon (He et al., 2012; Round et al., 2013; Hou et al., 2015). Throughout different historical periods and cultural traditions, auricular acupuncture has held significant prominence, with its positioning and role in TCM theory rendering it an indispensable component of Chinese medicine. The Yellow Emperor’s Inner Canon states: “The ear is the master of all organs; the five zang and six fu organs communicate and circulate through it.” Further elaborating on this, Sun Simiao of the Tang Dynasty in his Essential Formulas Worth a Thousand Gold Pieces notes: “Within the ear, there are acupoints located on the horizontal beam at the ear orifice. Acupuncture and moxibustion applied here treat diseases such as jaundice, hepatitis, and various infectious diseases.” This indicates that ancient physicians recognized the close connection between auricular acupoints and the internal organs of the body. Over the course of history, auricular acupuncture has evolved into a distinct medical system. Particularly, in medical classics such as the Prescriptions Worth a Thousand Gold Pieces and the Great Compendium of Acupuncture and Moxibustion, the discourse on auricular acupuncture is rich and thorough. Through long-term clinical practice, ancient medical practitioners observed that sensitive areas on the auricle, ear lobule, and ear tragus were linked to specific symptoms. As a result, they gradually developed auricular therapy as a distinct medical system, using specific points on the ear for the diagnosis and treatment of various ailments (Hou et al., 2015). Since modern times, with advancements in technology and increasing health awareness, various schools of auricular acupuncture have emerged, leading to a flourishing and competitive landscape. For example, Paul Nogier’s theory of auricular acupuncture from France was localized in China, giving rise to the Huang, Guan, and Chen-Xu schools. Additionally, there is the Yuchi school, which advocates for the micro-meridian theory of the ear, and the Xuanyuan school, which asserts the independent existence of ear meridians. Different countries and regions have published a variety of radically different auricular maps, and different schools of thought, including France, Germany, and China, have developed their own ear maps, resulting in inconsistent point locations. This inconsistency poses a challenge to both clinical practice and scientific research, so the diversity of auricular point maps and the lack of consensus on auricular point location are important problems in the field of auricular acupuncture. Wirz-Ridolfi emphasized the importance of precise positioning of auricular needles, emphasizing the need for standardization. Recent advances in near-infrared spectroscopy (NIRS), functional magnetic resonance imaging (fMRI) and optogenetics, beginning to bridge the gap between traditional auricular acupuncture and modern knowledge of anatomy and physiology. These techniques have the potential to validate auricular points and improve the accuracy of auricular point localization. The effectiveness and reliability of auricular acupuncture can be enhanced by correlating auricular acupoints with modern anatomical and physiological data (Wirz-Ridolfi, 2019).

The ear acupoint, as a representative therapeutic point in traditional Chinese medicine, has attracted widespread interest in modern medicine (Ernst, 2007). Advancements in modern medical technology have enabled scientists to observe and measure the physiological characteristics of the ear acupoint more accurately, such as blood circulation, neural conduction, etc., thereby discovering potential connections between information related to pathogens and body parts with certain areas of the earlobe (Wang et al., 2001). In 1956, the father of auricular acupuncture in France, Paul Nogier, used acupoint detectors to summarize the theory of somatotopic arrangement of the body on the earlobe (Gori and Firenzuoli, 2007), which later evolved into non-invasive electrical stimulation, light stimulation, and pressure therapy. After being introduced to China, Chinese acupuncture practitioners innovated and enriched it based on classical Chinese medicine theories, designing methods such as intradermal needles, ear-warming needles, ear-electro needles, and ear-water needles (Wirz-Ridolfi, 2019), providing a more accurate basis for its clinical application. Through continuous clinical exploration, Richard Niemtzow, based on Dr. Paul Nogier’s ear acupoint map and guided by modern medical theory, proposed the unique combination of ear acupoints for Battlefield Acupuncture, dynamically stimulating the cingulate gyrus, thalamus, Omega 2, point zero, and Shen Men with specially designed needles, which has been applied in the US military with good analgesic effects (King et al., 2013; Federman et al., 2018; Niemtzow, 2018). Furthermore, modern medical research on ear acupoints has gradually expanded to the molecular level. By analyzing gene expression and protein composition in ear acupoint tissues, researchers attempt to elucidate the molecular mechanisms of ear acupuncture therapy, providing a more scientific explanation for its role in treating certain specific diseases. For instance, recent studies have demonstrated that auricular acupuncture can modulate the expression of certain neurotransmitters, such as β-endorphin and serotonin, which are involved in pain regulation. Additionally, molecular research has shown that stimulation of specific ear acupoints can influence inflammatory pathways by altering cytokine levels, such as TNF-α and IL-6. These findings provide a deeper understanding of the mechanisms underlying the therapeutic effects of auricular acupuncture and highlight its potential for treating various conditions (Liao et al., 2017; Liao et al., 2018). This series of studies not only deepens the understanding of ear acupuncture therapy but also lays a solid foundation for its integration into the framework of modern medicine.

Anatomical studies have shown that the ear is the only part of the body surface reached by the vagus nerve (Peuker and Filler, 2002; Chen et al., 2015), which is referred to as the “great guardian of the human body,” maintaining the intricate neuroendocrine balance within (Yuan and Silberstein, 2016). The entire region of the ear acupoint forms a complex and delicate network of neurovascular systems connected to the central nervous system, such as the brain and spinal cord, facilitating close communication with internal organs through neural transmission and blood circulation. This mechanism of neural transmission renders the ear acupoint area a sensitive reflex zone capable of regulating multiple peripheral organs (Wang Y. et al., 2021), thus, ear acupoint stimulation is recognized for its application in various diseases associated with the central and peripheral nervous systems (Yu et al., 2021). Subsequently, researchers discovered that vagus nerve stimulation (VNS) is an implantable form of neuroregulation (George et al., 2000), which, due to its favorable performance in neurotransmitter and psychoneurological regulation domains (Groves and Brown, 2005), was approved by the US Food and Drug Administration (FDA) in 2005 for treating refractory depression (Aaronson and Conway, 2018). However, the high medical costs and traumatic nature of the treatment for patients have limited the development of VNS (Kong et al., 2018). Through subsequent research, scholars found that the physiological effects induced by stimulating the main branch of the vagus nerve located in the human ear are similar to VNS (Yakunina et al., 2017; Badran et al., 2018b; Badran et al., 2018c), leading to the derivation of transcutaneous auricular vagus nerve stimulation (taVAS) (Peuker and Filler, 2002; Badran et al., 2018a). As research progresses, the non-invasiveness (Austelle et al., 2022), efficacy, and broad applicability of taVAS have garnered academic attention and spurred discussions in medical practice (Badran et al., 2018b; Badran et al., 2019; Wang et al., 2022a; Wang et al., 2022b).

In clinical practice, auricular acupuncture has been widely utilized in the treatment of various diseases, accumulating a significant body of clinical trials and research (Vieira et al., 2018). These studies have demonstrated the significant therapeutic effects of auricular acupuncture in disease management and health promotion (Supplementary Table 1). However, despite the abundant clinical evidence supporting its clinical application, research on the mechanisms of action of auricular acupuncture remains relatively scarce. Existing studies have primarily focused on clinical outcomes without delving deeply into the underlying biological processes. For instance, while numerous trials have demonstrated the efficacy of auricular acupuncture in pain management, stress reduction, and treating various chronic conditions, the specific neural pathways, receptor interactions, and molecular changes involved are not well understood. Furthermore, many studies lack rigorous experimental design, such as adequate control groups, blinding, and standardized protocols, which limits the reproducibility and generalizability of their findings. There is a significant gap in understanding the dose-response relationships and the long-term effects of auricular acupuncture. Additionally, there is a need for more advanced imaging and biomarker studies to elucidate the physiological and biochemical changes induced by auricular acupuncture. Addressing these gaps is crucial for validating the therapeutic mechanisms and integrating auricular acupuncture into mainstream medical practice more effectively. This phenomenon has sparked widespread attention and research interest. Understanding the mechanisms of action of auricular acupuncture not only deepens our understanding of its clinical application but also helps provide scientific evidence for its further promotion in modern medicine. The purpose of this study is to provide a more comprehensive scientific basis for the rational application of auricular acupuncture in clinical practice by reviewing relevant literature and summarizing its mechanisms of action.

## 2 Anatomy of the ear

Auricular acupoints are unique therapeutic points in traditional Chinese medicine, characterized by a complex and subtle anatomical structure (Oleson, 2002). Through a thorough investigation of the anatomical features of the auricle, ear lobule, and other regions, we can gain a more comprehensive understanding of the mechanisms of action of auricular acupoints in medicine, aiming to unveil their true mysteries in medical practice.

The auricle constitutes a crucial region within the ear, comprising structures such as the helix, antihelix, tragus, antitragus, concha, and lobule. The anterior surface of the auricle is characterized by irregularities, while the posterior aspect tends to be relatively smooth with slight protrusions. The skin covering the auricle is rich in sensory nerve endings, rendering it highly sensitive to various stimuli such as pain and temperature (Silva et al., 2017). Studies have confirmed that unlike other areas of the body’s skin, specific representative areas exist within the somatosensory cortex for the auricle, including regions of the face, head, and neck (Nihashi et al., 2001). These differences in sensory perception and representative cortical areas hold significant implications for auricular acupuncture therapy. The main framework of the auricle is cartilaginous, exhibiting complex and variable shapes and structures. The unique properties of cartilage afford the auricle both elasticity and relative stability. This structural characteristic provides support and feedback during stimulation of auricular acupoints. The internal region of the auricle is richly supplied with nerves and blood vessels, located superficially within a gap of approximately 1 mm–1.5 mm between the auricular cartilage and the skin (Bermejo et al., 2017). The distribution of nerves primarily includes five branches: the auricular branch of the vagus nerve, originating from the cervical ganglion and crossing with fibers of the glossopharyngeal nerve, which then bifurcates into two branches—one distributed posteriorly and the other distributed anteriorly in the upper portion, including the concha and triangular fossa; the auriculotemporal nerve, derived from the mandibular branch of the trigeminal nerve, distributed in the anterior upper portion, including the helix; in addition, there are branches from the cervical plexus, the great auricular nerve, the lesser occipital nerve, as well as branches from the facial nerve and glossopharyngeal nerve, distributed posteriorly, including the helix and antitragus.

The innervation of the ear plays a critical role in the mechanisms underlying auricular acupuncture. Specifically, the auricular branch of the vagus nerve (ABVN), the trigeminal nerve, and the great auricular nerve are key players in this process. The ABVN innervates parts of the external ear and has connections to the nucleus tractus solitarius in the brainstem, which is involved in autonomic regulation and pain modulation. The trigeminal nerve, which innervates the anterior and superior regions of the auricle, connects to the trigeminal nucleus and the thalamus, influencing sensory processing and pain perception. The great auricular nerve innervates the lower part of the ear and is linked to cervical spinal nerves, contributing to the modulation of sensory and pain signals. These neural pathways highlight the intricate connections between the ear and the central nervous system, offering a neurological basis for the effects observed in auricular acupuncture. For example, the Shenmen point is located at the lower edge of the crus of the helix, near the lateral edge of the triangular fossa. This region is closely associated with a rich distribution of nerves. The Shenmen point is primarily innervated by the auriculotemporal nerve and the auricular branch of the vagus nerve. The auriculotemporal nerve, a branch of the trigeminal nerve, is responsible for transmitting sensory information from the auricle and surrounding areas. The auricular branch of the vagus nerve originates from the main trunk of the vagus nerve within the cranium and passes through the jugular foramen, extending along the ear canal to the anterior part of the auricle and the external auditory canal. The innervation of the Shenmen point is complex and is closely related to the functions of the autonomic nervous system, particularly in regulating heart rate, blood pressure, and digestive functions. Stimulation of the Shenmen point can influence the central nervous system through the vagus nerve, thereby exerting calming effects, modulating mood, and alleviating pain. These neural pathways interact to make the Shenmen point a key acupoint in clinical practice for treating anxiety, insomnia, pain, and various other conditions. The arterial supply consists primarily of two vessels: the superficial temporal artery supplying the anterior auricle and the posterior auricular artery supplying the posterior auricle. Additionally, the vascular system of the auricle includes corresponding veins (Imanishi et al., 1997). These nerves and blood vessels are interconnected with the entire nervous and circulatory systems, forming a complex network. The vascular system within the auricle not only provides ample blood supply to auricular acupoints but also plays a vital role in temperature regulation and nutrient delivery.

The tragus constitutes another significant region within the ear, characterized by a dense distribution of hair follicles that are associated with the growth and distribution of hair (Madaan et al., 2018). These hair follicles are not only involved in local sensation but also closely interconnected with the surrounding nervous and vascular systems (Prost-Squarcioni, 2006). Sebaceous glands are densely distributed on the tragus, secreting sebum to protect the skin and regulate moisture balance, thus maintaining the local skin’s hydration and softness (Geueke and Niemann, 2021). Sweat glands also play a crucial role in the anatomical structure of the tragus. Through the secretion of sweat, sweat glands participate in temperature regulation, as well as detoxification and the maintenance of skin cleanliness (Diao et al., 2019).

The lobule is an easily observable region within the ear, devoid of cartilage and composed of connective tissue and fat, rendering it relatively flat in shape. This feature makes the lobule an important stimulation point in auricular acupuncture therapy. Its anatomical structure includes blood vessels and nerves, among others. Stimulation of the lobule can directly affect the internal nervous and vascular systems of the auricle. The vascular system on the lobule is rich, with branches of the anterior auricular artery from the superficial temporal artery and perforating branches from the posterior auricular artery distributed throughout. This not only provides ample blood supply to auricular acupoints but also supports the overall blood circulation in the ear region, contributing to the unique physiological effects of the lobule in auricular acupuncture therapy (Park and Roh, 2002). The lobule is innervated by the great auricular nerve, and its nerve endings are connected to the central nervous system (Werner et al., 2021), forming an important reflex zone. Stimulation of the lobule can induce various physiological effects, including analgesia and relaxation.

While the anatomical study of the ear provides valuable insights into the mechanisms of auricular acupuncture, there are several limitations and challenges that must be considered. Individual variations in ear anatomy can lead to differences in the precise location and effectiveness of acupoints, which may impact clinical outcomes. Moreover, the small and complex structures within the ear present significant challenges for visualization, particularly with current imaging techniques such as MRI and CT scans. These techniques may lack the resolution necessary to capture fine details, potentially limiting the accuracy of anatomical studies. Addressing these challenges through the development of more advanced imaging technologies and standardized methodologies is crucial for advancing our understanding of auricular acupuncture and its clinical applications.

## 3 Traditional Chinese medicine classics and auricular acupoints

TCM is a comprehensive medical system with a long history, encompassing millennia of practical experience and medical wisdom. Within the TCM classics, auricular acupoints are extensively discussed and explored as a unique therapeutic point, delving into their positioning and applications. An in-depth examination of the TCM classics regarding auricular acupoints reveals the profound essence of this ancient therapy, thereby demonstrating its inherent value in modern medicine.

### 3.1 The status of auricular acupoints in TCM classics

The Yellow Emperor’s Inner Canon (Huangdi Neijing) is one of the classic works of TCM, encompassing theories and practices from ancient medicine. It records, “The ear is where the Conception Vessel pulses converge,” describing auricular acupoints as reflective areas on the body’s surface that are interconnected with various organs and tissues of the body. The “Observation of the Ear” section in the Great Compendium of Acupuncture and Moxibustion and the Correction of Massage Techniques further detail the specific locations of internal organs on the ear: “the upper part of the ear corresponds to the heart, the lower part corresponds to the kidneys, the back of the ear inside corresponds to the lungs, the back of the ear outside corresponds to the liver, and the middle of the back of the ear corresponds to the spleen.” This concept of using auricular acupoints as a means to regulate internal organs permeates throughout TCM theory. Specifically, different regions of the auricle correspond to different parts of the body, such as the earlobe corresponding to internal organs and the ear helix corresponding to the limbs. By carefully observing the morphology and color of the auricle, TCM practitioners can assess the patient’s physical condition and make differential diagnoses. This perspective laid the foundation for subsequent auricular acupuncture treatments, emphasizing the close connection between auricular acupoints and the body’s internal organs.

In traditional Chinese medicine theory, the meridian system serves as the pathway for the circulation of qi and blood throughout the body. Auricular acupoints, classified as acupoints, are believed to be connected with the meridians (Sator-Katzenschlager and Michalek-Sauberer, 2007; He et al., 2012). The Classic of the Yellow Emperor’s Inner Canon (Lingshu: Nine Palaces and Eight Winds) mentions the distribution of meridians in the ear, emphasizing the method of regulating qi and blood and balancing yin and yang through stimulating the earlobe. This perspective underscores the significance of auricular acupoints as a means of regulating overall qi and blood circulation.

### 3.2 Application of auricular acupoints in traditional Chinese medicine classics

Traditional Chinese medicine classics outline various therapeutic techniques for auricular acupoints, including massage, moxibustion, and acupuncture (Hou et al., 2015). The Synopsis of the Golden Chamber (Jin Kui Yao Lue) records external treatments such as ear blowing and cupping, which, through different methods of stimulating corresponding auricular acupoints, can regulate organ functions to treat visceral diseases. Take the auricular tip acupoint, for example, since the Tang Dynasty in China, its stimulation has mainly been through moxibustion, believed to harmonize yin and yang and promote blood circulation while dispelling stasis. The application of these therapeutic methods requires physicians to flexibly adapt them according to the specific conditions of patients to achieve the desired effect of balancing the body. The Great Compendium of Acupuncture and Moxibustion (Zhen Jiu Da Cheng) specifically addresses the “Ear and Eye Gateway,” detailing auricular acupoints’ treatment of related diseases, such as using the auricular tip and middle to treat cataracts and tinnitus. An analysis of these diseases reveals the outstanding performance of auricular acupoints in treating clinical conditions, providing crucial guidance for clinical practice. In addition to treating diseases, traditional Chinese medicine classics also emphasize the preventive and health-promoting roles of auricular acupoints. The Yellow Emperor’s Inner Canon (Huangdi Neijing) records, “Observe the ears to know one’s nature,” while the Correction of Massage Techniques (Li Zheng An Mo Yao Shu) dedicates a section to “Observation of the Ear,” indicating that ancient people inferred the severity of diseases by observing patients’ ears to facilitate early prevention. Regular massage and stimulation of auricular acupoints can regulate qi and blood, enhance constitution, and prevent disease occurrence. This holistic health concept is fully reflected in traditional Chinese medicine classics.

## 4 Innovative research in modern medicine

With the rapid development of scientific technology, research on auricular acupoints in modern medicine has entered a new era. Spanning multiple domains including neural regulation, pain management, mental health, metabolic disorders, and immune modulation, innovative research on auricular acupoints is bringing new insights and treatment modalities to the medical field.

### 4.1 Research on auricular acupuncture in neural regulation

Research in modern neuroscience has discovered that attempting to alter the nervous system can yield broad-ranging therapeutic effects, giving rise to a novel treatment strategy known as neural regulation therapy (Rossi et al., 2016). The neural reflex mechanisms within the auricular region have emerged as a notable area of innovative research.

#### 4.1.1 Analysis of neural reflex mechanisms

The vagus nerve, the longest cranial nerve in the human body, serves as a vital conduit connecting the brain to various organs and plays a crucial role in the expression and treatment of diseases. Despite constituting only a small portion of the nervous system, the vagus nerve exerts supervisory control over many essential physiological functions. Comprising 80% sensory neurons and 20% motor neurons (Foley and DuBois, 1937), vagal neurons extend bidirectional pseudounipolar axons, projecting to the brain on one end while innervating numerous major organs and tissues, including the heart, lungs, pancreas, and gastrointestinal tract, on the other. This bidirectional connection pervades the physiology and pathology of the human body (Prescott and Liberles, 2022). The vagus nerve is a mixed nerve containing five types of fiber components. These include general visceral motor fibers, special visceral motor fibers, general visceral sensory fibers, special visceral sensory fibers, and general somatic sensory fibers, which are closely associated with the dorsal nucleus of the vagus nerve, the nucleus ambiguus, the inferior part of the nucleus of the solitary tract, the superior part of the nucleus of the solitary tract, and the spinal trigeminal nucleus, respectively. The special visceral motor fibers innervate the striated muscles of the pharynx and larynx. General visceral motor fibers, after synapsing in parasympathetic ganglia within or near the organs, issue postganglionic parasympathetic fibers that distribute to the smooth muscles, cardiac muscles, and glands of the thoracic and abdominal organs. The cell bodies of general visceral sensory fibers are located in the inferior ganglion below the jugular foramen, with peripheral projections distributed to the organs in the thoracic and abdominal cavities. The cell bodies of general somatic sensory fibers are located in the superior ganglion within the jugular foramen, with peripheral projections distributed to the dura mater, auricle, and external ear canal.

**FIGURE 1:**
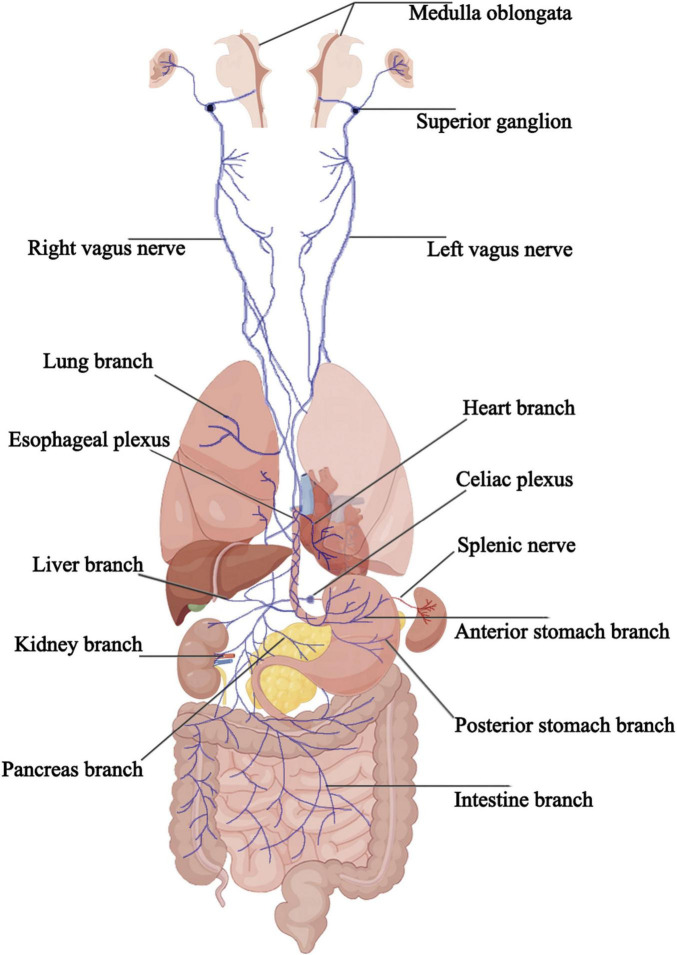
The connection between the vagus nerve and the various organs.

The vagus nerve exits the brainstem from the posterior part of the medulla oblongata, below the roots of the glossopharyngeal nerve, passing through the jugular foramen and containing the superior and inferior ganglia at its exit. It then descends behind the internal carotid artery and internal jugular vein, entering the thoracic cavity through the superior thoracic aperture. In the thorax, the paths and positions of the left and right vagus nerves differ. The bronchial branches, esophageal branches, and cardiac branches are small branches of the vagus nerve in the thorax that join the pulmonary plexus, esophageal plexus, and cardiac plexus, respectively. After descending into the thoracic cavity, the left vagus nerve primarily forms the left pulmonary plexus and the anterior esophageal plexus, which converge into the anterior vagal trunk at the lower part of the esophagus, entering the abdominal cavity and eventually branching into the anterior gastric and hepatic branches. The right vagus nerve, after descending into the thoracic cavity, primarily forms the right pulmonary plexus and the posterior esophageal plexus, which converge into the posterior vagal trunk at the lower part of the esophagus, entering the abdominal cavity and branching into the posterior gastric and celiac branches. The anterior and posterior gastric branches primarily run along the lesser curvature of the stomach, innervating the stomach wall near the lesser curvature; the hepatic branch follows the hepatic artery within the lesser omentum, forming the hepatic plexus and innervating the bile ducts and liver. The celiac branch is relatively larger and forms the celiac plexus near the celiac trunk, connecting with the spleen via the splenic nerve. In the abdomen, other smaller branches of the vagus nerve travel along the celiac plexus trunk, superior mesenteric artery, and gastric artery to reach the pancreas, small intestine, left colic flexure, and parts of the large intestine, kidneys, and adrenal glands. The cervical segment of the vagus nerve contains all five fiber types, the thoracic segment mainly comprises general visceral efferent fibers, general visceral afferent fibers, and special visceral efferent fibers, and the abdominal segment predominantly consists of general visceral efferent fibers and general visceral afferent fibers (Figure 1).

In-depth monitoring of neuronal activity in regions such as the auricle, earlobe, and ear tragus has revealed the intricate neural network and delicate regulatory mechanisms present in the auricular region, which serves as the sole cutaneous site innervated by the vagus nerve. This understanding has led to the concept of taVNS, wherein stimulation of the cutaneous sensory area innervated by the auricular vagus nerve is employed for disease treatment. Over the past two decades, transcutaneous auricular vagus nerve stimulation has emerged as a research hotspot for alternative therapies in various diseases, offering potential drug-free treatment modalities (Wang Y. et al., 2021).

#### 4.1.2 Changes and regulation of neurotransmitters

At the beginning of the 20th century, neuroscientists and physiologists identified acetylcholine as the first neurotransmitter associated with the vagus nerve (Loewi, 1956). With the gradual advancement of modern medicine, the discovery of the significant impact of auricular vagus nerve stimulation on neurotransmitter release has emerged (Thi Mai Nguyen et al., 2020). Through the application of molecular biology techniques, imaging technologies, and other means, researchers have found that auricular stimulation can modulate various neurotransmitters, such as catecholamines, β-endorphins, glutamate, and serotonin, thus playing a crucial role in neural regulation (Li et al., 2021; Wang H. et al., 2021). This finding provides new theoretical support for the application of auricular acupuncture in neurological disorders.

#### 4.1.3 Neuroplasticity and auricular acupuncture

Neuroplasticity is a complex phenomenon and a significant characteristic of the nervous system (de Oliveira, 2020). Relevant studies have found that vagus nerve stimulation can promote the release of neurotransmitters, such as norepinephrine, and enhance the gene expression of brain-derived neurotrophic factor and basic fibroblast growth factor, mediating neuroregeneration in emotional areas such as the hippocampus (Duman, 2004). Thus, auricular stimulation is considered capable of modulating neuroplasticity. By employing functional near-infrared spectroscopy, researchers have observed changes in the neural network structure and reorganization function of the brain following auricular acupuncture stimulation. This provides a new explanation for the efficacy of auricular acupuncture in neuroplasticity-related disorders (Zhang J. et al., 2023).

### 4.2 Innovative research on auricular acupuncture in pain management

#### 4.2.1 Regulatory mechanisms of pain signal transmission

**FIGURE 2:**
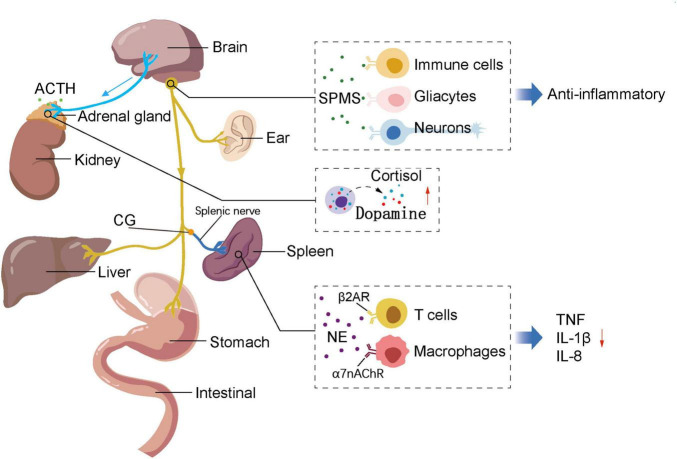
Hypothesis on the mechanism of auricular acupuncture for analgesia. After auricular acupuncture stimulation, the vagus nerve transmits signals to the celiac ganglion, triggering the release of acetylcholine, which in turn stimulates the splenic nerve to release norepinephrine. This neurotransmitter binds to T cell β2 adrenergic receptors and macrophage α7nAchR receptors, resulting in reduced cytokine production. Additionally, auricular stimulation induces the brain to release adrenocorticotropic hormone, which acts on the adrenal glands, promoting the production of cortisol and dopamine. Efferent adrenal gland innervation originates from the spinal cord and celiac plexus or afferent innervation of the adrenal glands synapse in the spinal cord (via dorsal root ganglia) and the vagal solitary tract nucleus (located in the MO). Furthermore, auricular stimulation leads to the generation of SPMs, which bind to their respective receptors and are expressed on immune cells, glial cells, and neurons, thereby modulating inflammation. ACTH, adrenocorticotropic hormone; SPMs, including lipoxins, resolvins, protectins, and maresins, are endogenous lipid mediators generated during the resolution phase of inflammation; CG, celiac ganglion; MO, medulla oblongata; NE, norepinephrine; β2 AR, β2 adrenergic receptor; α7nAChR, α7 nicotinic Ach receptor; IL-1β, Interleukin-1β; IL-8, Interleukin-8.

According to the International Association for the Study of Pain, pain is an unpleasant sensory and emotional experience associated with actual or potential tissue damage (Raja et al., 2020). Data studies indicate that up to 70% of emergency department visits are due to pain (Todd et al., 2007), and the global prevalence of chronic pain reaches 24% (Mansfield et al., 2016) Innovative research on auricular acupuncture in pain management primarily focuses on modulating pain signal transmission through the cholinergic anti-inflammatory pathway and the hypothalamic-pituitary-adrenal axis, by mediating the release of inflammatory cytokines (such as IL-1β, IL-8, and TNF-α), specialized pro-resolving mediators [SPMs, which consist of molecules derived from ω-3 fatty acids, including resolvins, protectins, and maresins, along with lipids originating from arachidonic acid (ω-6). These substances are involved in reducing inflammation, eliminating microbes, relieving pain, and supporting tissue regeneration through innovative pathways], and the expression of neuropeptides (Tracey, 2002; Lerman et al., 2016; Chaudhry et al., 2019; Shao et al., 2023; Figure 2). Cytokines are small peptide proteins secreted mainly by immune cells (Przemioslo and Ciclitira, 1996), exerting their effects via specific receptors on cell surfaces to facilitate intercellular communication, stimulate proliferation of antigen-specific effector cells, and mediate local and systemic inflammation (Neuman, 2007; Sanchez-Munoz et al., 2008), influencing pathological tissues and nerves, thereby generating pain (Alam and Chen, 2023). Through clinical observation and experimental studies, researchers have found that auricular acupuncture stimulation can activate the descending pain inhibitory pathway located in the spinal dorsal horn neurons, thereby modulating pain signal transmission and alleviating pain sensation (Slade et al., 2011). This phenomenon not only provides empirical evidence for the application of auricular acupuncture in postoperative and chronic pain fields but also offers insights for the development of novel pain management approaches (Usichenko et al., 2017).

#### 4.2.2 The association between auricular acupuncture and the gate control theory of pain

In 1905, Henry Head proposed the precursor to the Gate Control Theory of Pain by demonstrating through self-experimentation that the perception of pain from harmful stimuli could be alleviated by the transmission of non-harmful stimuli (Duan et al., 2018). In 1965, Ronald Melzack and Patrick Wall elucidated the processing of sensory input by the spinal cord through a series of animal experiments and clinical observations, identifying a simple pain coding mechanism and pioneering novel treatment approaches for pain. This mechanism, which encodes the harmful components of skin sensory input, came to be known as the Gate Control Theory of Pain (Mendell, 2014). According to this theory, primary nociceptive neurons (pain receptors) in the dorsal root ganglia (DRG) can detect harmful information to the body and transmit it to the brain through projection neurons in the spinal dorsal horn (DH) (Basbaum et al., 2009), Some inhibitory interneurons in the DH (gate control neurons) can reduce the transmission of input from large-diameter touch fibers and small-diameter pain fibers, thereby inhibiting the transmission of pain and touch pathways to DH nociceptive projection neurons (Mendell, 2014; Cui et al., 2016). Sensory afferents from the rat tragus have been observed to project primarily to the dorsal horn of the upper cervical spinal cord and, to a significant extent, to the Pa5 region of the brainstem, with lesser projections to the nucleus tractus solitarius (NTS). Tragus stimulation led to sympathoinhibition, as recorded from the lumbar sympathetic chain. The neuronal tracing results showed substantial innervation of the upper cervical cord by afferents originating from the tragus. When the C1-C3 ipsilateral nerves were transected in working heart brainstem preparation, the observed loss of sympathoinhibition suggests that the reduction in sympathetic nerve activity was at least partially mediated through these projections. This highlights the crucial role of sensory afferent pathways in influencing autonomic and pain responses, which is consistent with the principles of the gate control theory, especially in the context of auricular acupuncture (Mahadi et al., 2019). Auricular stimulation, by activating large-diameter fibers, can close the transmission pathway of pain signals, thereby modulating the perception of pain through the Gate Control System (Xu et al., 2021). Modern researchers have further elucidated the mechanisms of auricular stimulation’s analgesic effects by thoroughly analyzing its impact on the Gate Control System of Pain (Dean and Geiringer, 1990). The cholinergic anti-inflammatory pathway and the Gate Control Theory both play significant roles in pain modulation. However, research exploring the direct relationship between these two mechanisms remains limited, highlighting a gap in the current understanding of how they may interact within the broader context of pain regulation. Future research should aim to bridge this gap, potentially uncovering novel insights into their combined effects on pain management.

#### 4.2.3 Relationship between brain activity and pain alleviation by auricular acupuncture

**FIGURE 3:**
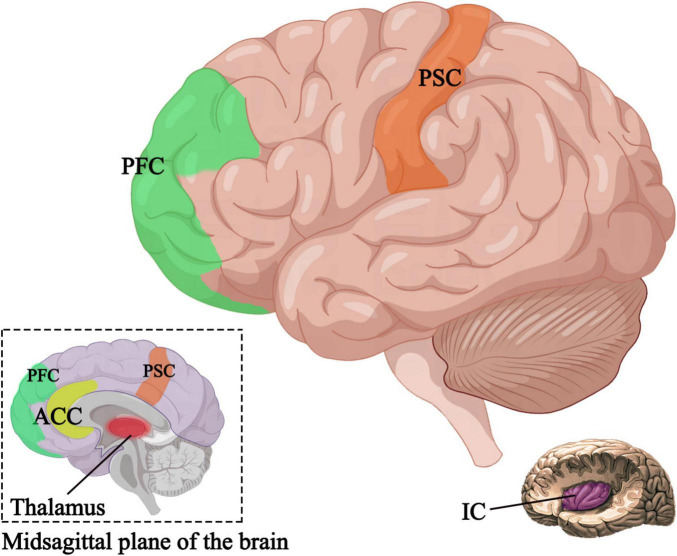
Active brain regions after auricular acupuncture. PSC, primary somatosensory cortex; IC, insular cortex; ACC, anterior cingulate cortex; PFC, prefrontal cortex.

Research has shown that the primary somatosensory cortex, insular cortex, anterior cingulate cortex, prefrontal cortex, and thalamus are positively correlated with pain (Apkarian et al., 2005; Bentley et al., 2016). In patients experiencing pain, the major regions of the brain in an activated state are the bilateral insular cortex and secondary somatosensory cortex, which, when electrically stimulated, can induce pain sensation (Peyron, 2014). Harmless stimulation in these regions can lead to abnormal bilateral expansion (Peyron et al., 2013). Through the application of brain imaging techniques such as fMRI, studies have investigated the effects of auricular acupuncture stimulation on brain activity (Romoli et al., 2014; Badran et al., 2018b). It has been found that auricular stimulation can induce changes in the activity of brain regions associated with pain processing (Romoli et al., 2014; Zhang et al., 2021; Figure 3), and can even restore damaged gray matter (Ong et al., 2019), providing new insights into the brain mechanisms of auricular acupuncture in pain management. At the same time, in addition to relieving pain, some studies have found that auricular acupuncture can activate the primary motor M1 area of the brain through functional near-infrared brain functional imaging data collection, thereby improving the upper limb motor deficits of patients after stroke (Zhang J. et al., 2023).

### 4.3 Innovative research on auricular acupuncture in mental health

#### 4.3.1 Effects of auricular acupuncture treatment on anxiety and depression

According to diagnostic criteria, anxiety and depression are considered as two distinct disorders, yet they often co-occur clinically, presenting as a common syndrome (Choi et al., 2020). Approximately 85% of patients with depression exhibit significant anxiety, while 90% of those with anxiety disorders also experience depression (Tiller, 2013). In 2000, Rush conducted a study on VNS, revealing the significant potential of VNS in modulating emotions in severely depressed patients resistant to conventional medication (Rush et al., 2000). Between 2005 and 2017 alone, there were over 3,000 articles on VNS therapy for epilepsy or depression (Carreno and Frazer, 2017). Modern medical research on auricular acupuncture in the realm of mental health indicates that transcutaneous taVNS and implantable vagus nerve stimulation (iVNS) act on the same neural pathways (Assenza et al., 2017), with iVNS demonstrating efficacy specifically in depressed populations (Nicholson et al., 2017). Previous studies have confirmed the significant alleviating effects of auricular acupuncture on anxiety and depression (Hein et al., 2013; Trevizol et al., 2015; Trevizol et al., 2016; Wu et al., 2018). Through systematic clinical research and psychological experiments, researchers have utilized fMRI technology to discover that auricular stimulation can influence the emotional regulation center in the brain by mediating the default network in the limbic system (Fang et al., 2016; Garcia et al., 2021), thereby improving patients’ psychological state (Round et al., 2013; Austelle et al., 2022).

#### 4.3.2 Mechanisms of auricular acupuncture on the autonomic nervous system

In 1832, German anatomist Professor Friedrich Arnold discovered that stimulating the external auditory canal could induce coughing similar to the stimulation of the vagus nerve, thus suggesting a potential connection between auricular acupuncture and the autonomic nervous system (He et al., 2012), specifically the relative activity levels of the sympathetic and parasympathetic nervous systems. Relevant studies have demonstrated that the auricular vagus nerve can transmit nerve fibers through the nucleus of the solitary tract (NST) to various regions, including the reticular formation, pre-ganglionic neurons of the visceral parasympathetic ganglia, the paraventricular nucleus of the hypothalamus, the thalamus (visceral motor center), and the amygdala, thereby mediating various reflexes (Hou et al., 2015). Auricular stimulation, by increasing vagal nerve tone, modulates the balance of the autonomic nervous system, leading to reductions in heart rate and blood pressure, as well as increases in blood flow and heart rate variability (Lin et al., 2011). Non-invasive heart rate variability biofeedback can improve stress-related disorders, depression, and panic disorders (Blase et al., 2021), thereby providing a physiological explanation for the use of auricular acupuncture in treating psychological health disorders such as anxiety and depression and suggesting a new direction for personalized treatment plans.

#### 4.3.3 Improvement in sleep quality

Sleep disorders can occur across all age groups and have a significant impact on daily work and life (Yeung et al., 2012), with severe cases leading to mental health issues such as depression (Gong et al., 2021). For patients with sleep disorders, the main treatment options are pharmacotherapy and physical therapy. However, pharmacotherapy can have side effects such as affecting brain activity and dependency, leading sleep disorder guidelines to prioritize physical therapy as the preferred method for improving sleep quality (Qaseem et al., 2016; Krystal et al., 2019; Mysliwiec et al., 2020; Riemann et al., 2023). Auricular acupuncture for improving sleep quality has become a recent research focus. Through experimental observations and patient reports, researchers have found that the main pathological manifestation of sleep disorders is dysfunction in the prefrontal cortex (Zou et al., 2013; Dai et al., 2016; Li et al., 2016). Researchers found that auricular stimulation regulates neural excitability by promoting the projection of neurotensin in the forebrain and limbic system, thereby improving sleep quality (Shouse et al., 1996). It can also regulate melatonin secretion (Spence et al., 2004), improve sleep structure (Wang et al., 2009), and demonstrate significant therapeutic effects for insomnia patients (Suen et al., 2002; Sjöling et al., 2008; Wu et al., 2014), providing new evidence for the application of auricular acupuncture in sleep disorders.

### 4.4 Innovative research of auricular acupuncture in other fields

#### 4.4.1 Application of auricular acupuncture in metabolic disorders

**FIGURE 4:**
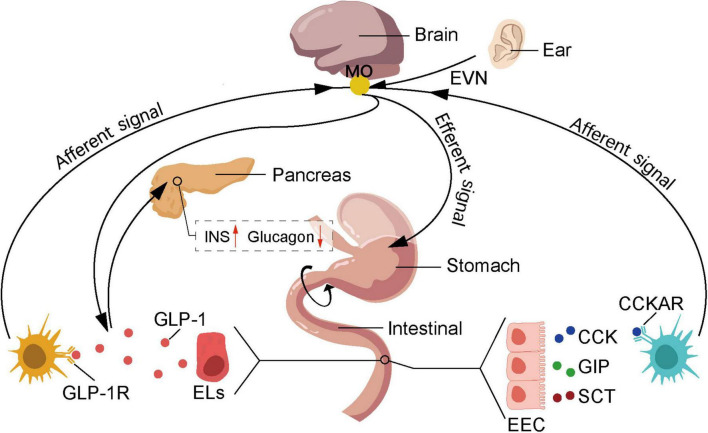
Auricular acupuncture affects feeding behavior by stimulating the vagus nerve. The nutrients absorbed by duodenal epithelial cells stimulate the secretion of cholecystokinin (CCK), glucose-dependent insulinotropic polypeptide (GIP), and secretin (SCT) by enteroendocrine cells (EECs), which in turn stimulates the secretion of glucagon-like peptide-1 (GLP-1) by enterochromaffin-like cells (ELs). GIP promotes insulin secretion by activating specific receptors on β-cells and acts on the hypothalamus to increase satiety. SCT can stimulate the secretion of pancreatic enzymes. CCK binds to cholecystokinin A receptors (CCKAR) on nerves in the gastrointestinal vagus, activating the vagus nerve to transmit information to the NST, thereby increasing satiety. Additionally, GLP-1 binds to receptors on hepatic portal vein vagus nerve, activating the vagus nerve, where the brain receives and integrates information and transmits it to the pancreas, thereby promoting insulin secretion and reducing glucagon secretion levels. NST, nucleus of the solitary tract; EVN, ear vagus nerve; EEC, enteroendocrine cells; Els, enterochromaffin-like cells; CCK, cholecystokinin; GIP, glucose-dependent insulinotropic peptide; SCT, secretin; GLP-1, glucagon-like peptide-1; CCKAR, cholecystokinin type A receptor; GLP-1R, glucagon-like peptide-1 receptor.

Innovative research on auricular acupuncture in the management of metabolic disorders has garnered significant attention (Suen et al., 2017). Studies have revealed that auricular stimulation can regulate insulin sensitivity (Li et al., 2019) and improve glucose metabolism (Zhang et al., 2020), offering new perspectives for the prevention and treatment of metabolic diseases (de Moraes et al., 2023). Metabolic disorders such as diabetes are on the rise globally, with the number of individuals diagnosed with type 2 diabetes alone reaching 400 million in 2016 (Zimmet et al., 2016). Moreover, it is estimated that by 2050, diabetes will affect one-third of the population in the United States (Boyle et al., 2010). According to the Global Diabetes Report, diabetes can lead to serious complications such as heart disease, kidney failure, blindness and lower limb amputations (van Dieren et al., 2010; ElSayed et al., 2023), posing a significant threat to patients’ health and wellbeing (Stratton et al., 2000). The etiology of diabetes is broad and complex (Rizza, 2010), resulting from the interplay of environmental and genetic factors (Smushkin and Vella, 2010), and can be summarized as either impaired insulin secretion or insulin resistance (DeFronzo, 1988). Relevant research has confirmed the close relationship between the auricle and diabetes, which can be utilized not only for diagnosis (Suen et al., 2015; Suen et al., 2017) but also for treatment (Boccino, 2023). Auricular vagus nerve stimulation mimics and accelerates the signaling of the intestinal vagus nerve, regulating satiety and glucose metabolism through feedback from the central nervous system (Figure 4). The intestinal vagus nerve does not directly contact intestinal substances but forms synapses with intestinal cells, acting as chemoreceptors that receive signals such as glutamatergic and serotonergic. After eating, enteroendocrine cells (EECs) in the gastrointestinal tract produce orexigenic peptides, which are transmitted to the nucleus tractus solitarius through the vagus nerve, reducing appetite (Richards et al., 2021). Vagal nerve activation promotes the secretion of glucagon-like peptide-1 (GLP-1) precursor by 30 amino acids, enhancing insulin secretion (Longo et al., 2023), thereby reducing blood glucose levels. However, it is worth to note that the increase of GLP-1 activates GLP-1 receptors through the vagus-dependent glucagon-like peptide-1 signaling pathway, stimulating the brain regions that are projected by the vagus nerve, thereby reducing the body’s locomotor capacity (Lai et al., 2024). On the other hand, the pancreas contains numerous origins of the vagus nerve. Selective chemogenetic activation of pancreatic β-cells elicits responses in the vagal sensory neurons of the nodose ganglion. Stimulation of the vagus nerve can activate pancreatic β-cells, leading to the production and release of serotonin from insulin granules in these cells. Serotonin contributes to the paracrine control of hormone secretion, promoting the functional state of the islets, thereby facilitating insulin stimulation and inhibiting glucagon release (Makhmutova et al., 2021).

#### 4.4.2 The role of auricular points in immune regulation

The role of auricular acupuncture in immune regulation is currently a focal point of research. Throughout their lives, humans face countless threats from pathogenic agents, and the immune system plays a crucial role in maintaining health and happiness by combating pathogens and maintaining internal homeostasis (Parkin and Cohen, 2001). The immune system comprises a network of cells distributed throughout the body (Shilts et al., 2022), which utilize interactions between cell surface protein groups to perform both defensive and surveillance functions (Rieckmann et al., 2017; Sattler, 2017). Auricular stimulation has been found to enhance the expression of tyrosine hydroxylase and α7 nicotinic acetylcholine receptors in the substantia nigra, leading to a reduction in levels of inflammatory cytokines and interleukins. Furthermore, auricular stimulation can increase the number of regulatory Treg cells (Jiang et al., 2018), thereby modulating innate immune responses and inhibiting the progression of inflammation (Badran et al., 2022; Go et al., 2022). Studies have demonstrated that auricular acupuncture positively impacts the balance of the immune system through pathways such as modulation of immune cell activity and influencing the secretion of immune mediators (Lin et al., 2015; Hesampour et al., 2023; Sahn et al., 2023).

## 5 Limitations

A significant limitation in the current study is the lack of standardized assessment tools to measure pain and treatment outcomes. The variability in assessment methods across different studies complicates the comparison of results and may obscure the true efficacy of auricular acupuncture. Establishing and utilizing standardized tools would allow for more reliable comparisons and better validation of the therapeutic effects of auricular acupuncture.

Moreover, while the benefits of auricular acupuncture have been emphasized, it is equally important to consider potential adverse effects and contraindications. Understanding the safety profile of auricular acupuncture is essential, particularly when treating populations with complex health issues. Adverse effects, though generally rare and mild, can include discomfort, infection, or dizziness, and certain conditions may contraindicate the use of auricular acupuncture (Tan et al., 2014). Future studies should prioritize the development of standardized safety guidelines and comprehensive assessments to ensure the safe and effective integration of auricular acupuncture into clinical practice (Fei et al., 2022).

While historical texts and traditional practices provide foundational insights into auricular acupuncture, addressing the scientific validation of this therapy is crucial. Recent reviews of randomized controlled trials indicate that, although some studies have reported positive outcomes, many others have failed to demonstrate significant efficacy compared to placebo treatments. This discrepancy suggests that the observed benefits may largely stem from placebo effects rather than the treatment itself. A detailed analysis of existing randomized controlled trials (RCTs) is necessary to provide a comprehensive understanding and to emphasize the need for further high-quality research to establish the efficacy of auricular acupuncture. Although traditional practices offer valuable context, modern scientific inquiry is essential for validating therapeutic claims. It is important to acknowledge the contributions of historical texts while also emphasizing the importance of rigorous scientific validation.

While various applications of auricular acupuncture have been explored, it must be recognized that not all conditions respond well to this treatment. The efficacy of auricular acupuncture is influenced by multiple factors, including the environment and the patient themselves, and invasive auricular acupuncture carries potential risks such as infection. Additionally, differences between physicians in point location, interpretation, stimulation methods (e.g., acupuncture or acupressure), and the practitioner’s level of experience may lead to inconsistent treatment results. This variation reflects a lack of standardized protocols in auricular acupuncture practice, which not only affects the reliability of clinical outcomes but also limits further development in the field. Therefore, a more in-depth discussion of auricular point mapping is needed to promote the development and adoption of standardized procedures to ensure the efficacy and reproducibility of auricular acupuncture.

The current text also lacks a comparative analysis of auricular acupuncture with other acupuncture modalities or alternative therapies, which prevents a full exploration of the unique advantages and limitations of auricular acupuncture within a broader therapeutic context. A comparative analysis would clarify the distinct aspects of auricular acupuncture, such as its effectiveness in specific symptoms or conditions, its ease of application, or its suitability for certain patient populations. This analysis would also help to identify its limitations, such as its potentially lower effectiveness compared to other therapies in certain cases. Including such a comparison would not only highlight the unique therapeutic effects of auricular acupuncture but also provide a more diverse range of treatment options for clinical practice.

Additionally, the manuscript does not sufficiently discuss potential biases and confounding factors that may affect the results of the studies reviewed. For instance, factors such as patient expectations, the experience level of practitioners, and the subjective nature of pain and psychological assessments can introduce significant variability in outcomes. This variability may impact the perceived efficacy of auricular acupuncture and should be addressed in future studies. Furthermore, the issue of publication bias, where studies with positive results are more likely to be published than those with negative or inconclusive findings, can create a skewed perception of auricular acupuncture’s effectiveness. Acknowledging and addressing these biases is crucial for a more accurate and balanced understanding of the treatment’s efficacy.

Finally, as part of traditional Chinese medicine, auricular acupuncture carries a rich cultural heritage. In the modern context, integrating this traditional practice into Western medicine necessitates addressing important cultural and ethical issues. One critical concern is the potential for cultural appropriation, where elements of traditional practice may be adopted without proper respect or understanding of their cultural significance. This can lead to the dilution or misunderstanding of the original practice, raising ethical concerns and issues of cultural insensitivity. Moreover, as auricular acupuncture becomes more widely adopted in Western healthcare systems, it is crucial to ensure that these practices are respected and responsibly integrated. This includes acknowledging the origins of the practice, respecting the cultures from which it arose, and engaging in dialog with practitioners from those cultural backgrounds. Ethical considerations also involve providing patients with accurate and evidence-based information, ensuring they are aware of the benefits and limitations of the treatment. Incorporating these cultural and ethical perspectives into the discussion will contribute to a more nuanced understanding of the role of auricular acupuncture in modern healthcare, emphasizing the importance of respecting the cultural origins of the practice while ensuring its ethical application in a contemporary medical environment.

## 6 Future prospects of auricular acupuncture

Innovative research on auricular acupuncture in modern medicine has provided a more scientifically grounded support for its application in clinical practice. Currently, auricular diagnostic stimulation techniques are becoming increasingly sophisticated and refined (Romoli et al., 2016), allowing for the diagnosis and treatment of diseases through resistance-sensitive points and pain-sensitive points (Kwai-Ping Suen et al., 2012; Romoli et al., 2016). In addition to traditional methods such as acupuncture and moxibustion, various approaches including massage, acupressure, and electrical stimulation are also employed. Different stimulation techniques can be individually selected based on the specific conditions of patients, thereby enhancing the specificity and comfort of treatment.

Auricular acupuncture, as a focal point of interest in the medical field, offers extensive research opportunities bridging traditional Chinese medicine theory and modern medical practices. With continuous technological advancements, further breakthroughs in auricular research are anticipated in the future, providing new perspectives and therapeutic modalities for the advancement of medical science. However, despite the significant potential of auricular acupuncture in medical research, it still faces a series of challenges, including the need for a deeper understanding of auricular mechanisms, standardization of clinical studies, and addressing individual variabilities in auricular treatment. Therefore, for a more effective utilization of auricular acupuncture in medicine, future research needs to delve deeper into the following areas.

### 6.1 In-depth study of the molecular mechanisms of auricular acupuncture

Although modern medicine has extensively researched the effects of auricular acupuncture, its specific molecular mechanisms and physiological basis still require further exploration. Future research could employ more sophisticated techniques, such as single-cell transcriptomics (a method for studying the gene expression of individual cells) (Ding et al., 2022) and proteomics (the large-scale study of proteins, their structures, and functions) (Hanash, 2003) to study the effects of auricular stimulation on gene expression, protein synthesis, and cellular signaling pathways. This will help uncover the cellular-level effects of auricular stimulation, providing a more detailed explanation for the interaction between the auricle and multiple systems including the nervous, immune, and endocrine systems. In addition, future studies should also emphasize and verify the research mechanism of auricular acupuncture in some specific diseases, such as obesity and smoking cessation. This requires us to make more efforts to improve the robustness of clinical trials, which will provide valuable insights into the development trajectory of auricular acupuncture research and ultimately provide a more scientific basis for future clinical practice (Chun et al., 2024).

To advance the understanding of the mechanisms of action of auricular acupuncture, future research should adopt more rigorous methodologies. Key recommendations include conducting pilot studies with sham controls to isolate the specific effects of auricular acupuncture, using larger and more diverse sample sizes to enhance statistical power and generalizability, and adopting standardized treatment protocols to ensure consistency and facilitate comparisons across studies. Additionally, improved trial designs, such as randomized controlled trials and longitudinal studies, are crucial for minimizing biases, assessing long-term effects, and ultimately leading to more effective integration of auricular acupuncture into clinical practice.

### 6.2 Application of auricular acupuncture in personalized medicine

Traditional medical practices typically rely on clinical symptoms, signs, and data such as gender, age, height, weight, family medical history, laboratory, and imaging assessments for diagnosis and treatment. This approach is relatively passive, as treatment often begins after symptoms appear. However, due to significant differences in individual genomic, proteomic, and metabolic profiles, diseases manifest and progress differently, making it challenging for clinicians to prescribe medication accurately. This limitation highlights the core goal of personalized patient care (Hamburg and Collins, 2010; Ho et al., 2020). Personalized medicine, or precision medicine, offers actionable health management based on extensive individual information (Goetz and Schork, 2018; Braig, 2022). The unique nature of auricular acupuncture presents vast prospects for its application in personalized medicine (Cirillo et al., 2022). By establishing large-scale individual databases and analyzing the physiological and psychological responses of different populations to auricular stimulation, scientific foundations can be provided for developing personalized auricular acupuncture plans. This will enable healthcare professionals to tailor auricular acupuncture more accurately to individual characteristics, thereby enhancing treatment precision, efficacy, and individualization (Kaniusas et al., 2019). Future research can integrate factors such as genetic background, lifestyle, and environment to explore the impact of individual differences on the effectiveness of auricular acupuncture. The effectiveness of auricular acupuncture can be significantly influenced by patient-specific characteristics such as age, gender, psychological state, and pain type. Younger and older patients may respond differently to treatment, while psychological factors like stress levels can modulate pain perception and therapy outcomes. Additionally, gender differences in pain sensitivity and response to acupuncture suggest the need for gender-specific approaches. The nature of the pain—whether acute or chronic—also impacts the choice of acupoints and stimulation techniques. Therefore, incorporating these factors into personalized treatment strategies is crucial for optimizing the therapeutic benefits of auricular acupuncture. Future research should further investigate these variables to enhance the efficacy of tailored acupuncture protocols.

### 6.3 Interdisciplinary integration of auricular acupuncture

With the trend of integration between traditional Chinese and Western medicine, auricular acupuncture is expected to be more closely integrated with modern medical techniques in the future. This integration aims to apply modern medical theories to guide clinical auricular treatment. By combining auricular acupuncture with pharmacological and surgical treatments across multiple disciplines, its advantages in multidisciplinary comprehensive treatment can be better utilized to enhance treatment outcomes (Gori and Firenzuoli, 2007; Zhao et al., 2012; Lin and Hsieh, 2014). For instance, by incorporating knowledge from neuroscience, psychology, and nutrition, a deeper exploration of the role of auricular acupuncture in neural regulation, psychological health, metabolic control, and the management of nausea and vomiting induced by cancer chemotherapy can be achieved (Paiva et al., 2024). Such interdisciplinary research efforts will contribute to the development of a more comprehensive theory of auricular acupuncture and provide innovative directions for multidisciplinary collaboration.

### 6.4 Prospects of auricular acupuncture in chronic disease management

Chronic disease management poses a significant challenge in the medical field. By establishing a rational doctor-patient relationship and conducting large-scale clinical studies to observe the effects of auricular acupuncture on physiological indicators and quality of life in patients with chronic diseases, more comprehensive evidence can be provided for the application of auricular acupuncture in chronic disease management, thereby enhancing the effectiveness of clinical healthcare (McGowan, 2012) and demonstrating its potential application prospects (Zeliadt et al., 2020). Future research can delve deeper into exploring the mechanisms of auricular acupuncture in the prevention and control of chronic diseases, particularly its potential in the comprehensive treatment of conditions such as diabetes, hypertension, and cardiovascular diseases.

### 6.5 In-depth research on auricular acupuncture in the field of neuroscience

Our current understanding of auricular acupuncture primarily centers on the role of the vagus nerve. However, there is a notable lack of detailed research on the involvement of other nerves, such as the trigeminal nerve, greater auricular nerve, and lesser occipital nerve, and their connections to the central nervous system. To address this gap, future studies should focus on elucidating the specific functions and pathways of these nerves in auricular acupuncture. Investigating their roles and interactions with the central nervous system will provide a more comprehensive understanding of the neurological basis of auricular acupuncture and enhance the development of targeted therapeutic strategies.

Furthermore, as our knowledge of neuroscience advances, future research should explore the intricate mechanisms underlying auricular acupuncture’s effects on neural regulation. Advanced neuroimaging techniques, including fMRI and electroencephalography, will enable researchers to monitor and analyze the real-time impact of auricular stimulation on brain activity, providing deeper insights into its therapeutic mechanisms (Yakunina et al., 2017; Butt et al., 2020; Sclocco et al., 2020; Zhang et al., 2021). By extensively establishing connectivity maps between auricular points and brain regions, researchers can gain a more comprehensive understanding of the overall impact of auricular acupuncture on the neural network. This holds the promise of providing more precise evidence for the customization of auricular acupuncture in the treatment of neurological disorders (Zhang et al., 2021).

### 6.6 Expanding the application scope of auricular acupuncture

Although auricular acupuncture has achieved significant success in areas such as neural regulation, pain management, and psychological wellbeing, its potential applications in other fields remain underexplored. Future research can further expand the application of auricular acupuncture in areas such as chronic disease management, metabolic disorders, and immune regulation. By validating its efficacy in a broader range of fields, a more solid foundation can be established for the comprehensive application of auricular acupuncture in medicine.

## 7 Conclusion

The auricle, as a unique and mysterious therapeutic locus in TCM, finds its foundation in a detailed anatomical analysis and the understanding of its intricate neurovascular structure. This microsystemic nature of the auricle, reflecting the whole body, forms the basis for its complex therapeutic effects. While classical TCM literature offers varied descriptions of auricular points, they collectively emphasize the close connection between auricular points and the body’s meridians and organs. Systematic examination reveals that traditional TCM theories provide insights for modern medical research, particularly in personalized healthcare and comprehensive disease management.

Building upon anatomical and classical theoretical foundations, we focus on innovative research on auricular acupuncture in modern medicine. This section encompasses the roles of auricular acupuncture in neural regulation, pain management, psychological wellbeing, metabolic disorders, and immune regulation. Auricular stimulation not only alleviates various pain symptoms but may also impact patients’ psychological wellbeing through neural regulation. Through in-depth exploration of modern medical research, we anticipate the potential applications of auricular acupuncture in future medical studies. Advancements in technology will allow for deeper insights into the molecular mechanisms underlying auricular acupuncture, providing a scientific basis for its application in personalized healthcare and chronic disease management.

In summary, in-depth exploration of auricular points in classical TCM literature provides guidance for their medical applications. From ancient auricular localization and treatment principles to modern scientific validation, auricular acupuncture boasts a long history and rich practical experience. With technological advancements, our understanding of auricular acupuncture will deepen, and future research will offer more scientific support for its extensive application in medicine. Through the integration of traditional wisdom and modern science, the unique medical element of auricular acupuncture will continue to contribute its distinctive value to human health. Despite significant progress in auricular research, its mysteries remain partly unsolved. Future research efforts will need to focus on addressing these challenges through in-depth mechanism studies, standardized clinical research, and exploration of individual differences, paving the way for broader applications of auricular acupuncture in medicine. Auricular acupuncture, this ancient and mysterious medical tradition, holds promise for rejuvenation in modern medicine, showcasing multifaceted potential therapeutic value and bringing forth new possibilities for human health.
