# Supplementary material for: Multi-level exploration of auricular acupuncture: from traditional Chinese medicine theory to modern medical application
Source: Front Neurosci. 2024 Sep 23;18:1426618. doi: 10.3389/fnins.2024.1426618 (PMC11456840; doi:10.3389/fnins.2024.1426618)
Supplement: Supplementary file 1 [file Table_1.docx]

**Table 1** Clinical application cases of auricular acupuncture RCT:randomized controlled trial

| **Disease** | **RCT/Study/RT** | **Sample Size** | **Control group** | **Acupoints** | **Frequency and duration of treatment** | **Ear needle apparatus** | **Conclusion** | **References** |
| --- | --- | --- | --- | --- | --- | --- | --- | --- |
| Cancer survivors with chronic musculoskeletal pain | RCT | 360 | Electroacupuncture, and usual care | Cingulate gyrus point, thalamus, omega 2, point zero, shen men | Each treatment was retained for 3-4 days, once a week, for a total of 10 treatments | ASP needle (Sedatelec) | Auricular acupuncture can significantly reduce the pain and improve the physical and mental health of patients | (Mao et al., 2021) |
| Cancer pain | RCT | 31 | A Placebo group, in which fixed placebo points were used | Shenmen, Kidney, Sympathetic, Muscle Relaxation and the energy balance points | Each treatment was retained for one week, once a week for a total of 8 weeks | Semi-permanent auricular needles, size 0.20 mm x 1.5 mm | Auricular acupuncture can significantly relieve cancer pain | (Ruela et al., 2018) |
| SupraSpinatus Tendinopathy | Study | 45 | Control group (The selected acupoints were not associated with SupraSpinatus Tendinopathy) | Shoulder pain / Resistance sensitive point, Pain sensitive point(areas SF1 and SF2) | Hold for 10 seconds | Seirin® type B needle 0.30 x 30 mm brown handle | Auricular acupuncture can effectively relieve shoulder pain | (Lovato et al., 2023) |
| The pain after cesarean delivery | RCT | 180 | The sham-auricular point group( without built-in needle ) | MA-SC, MA-TF1, MA-IC1 and MA-AT1 | Auricular acupuncture was placed before surgery and retained until the third postoperative day | New Pyonex needles, sized 0.2 x 1.5 mm | The use of preoperative acupuncture as an additional pain therapy is safe and effective in patients after elective cesarean delivery | (Usichenko et al., 2022) |
| The pain in patients with dementia | RCT | 206 | The sham-auricular point group( without semen vaccariae ) | Shenmen (TF4), subcortex (AT4), adrenal (TG2P), and two auricular points corresponding to pain sites | Each auricular point will be pressed gently 2 times lasting 1 min each time. After the whole intervention, these tapes will be removed. | Semen Vaccariae® (about 2 mm in diameter, Taicheng Technology and Development Co., Ltd., Shanghai, China) | Auricular acupuncture can effectively relieve the pain of patients and is more easily accepted by patients | (Chai et al., 2022) |
| The pain in patients with partial lung resection | RCT | 92 | The sham-auricular point group( without semen vaccariae ) and medication group(Atropine sulfate injection, Dezocine injection) | Shenmen (TF4), sympathetic (AH6a), subcortex (AT4), brain stem (AT3,4i), anterior ear lobe (LO4), chest (AH10) and lung (CO14) | Auricular acupuncture was placed before surgery and retained until the third postoperative day | Auricular point sticking with Wangbuliuxing | Auricular point sticking therapy relieves perioperative pain, shortens analgesic time, releases anxious and depressive emotions and reduces postoperative adverse reaction in the patients with partial lung resection. The analgesic mechanism is probably related to the increase of plasma concentration of β-endorphin. | (Li et al., 2021) |
| Chronic Low Back Pain | Study | 74 |  | Shenmen, nervous subcortex,the lumber vertebral area near the antihelix middle line, the upper 4/5 positive area (front and back ear area) | After auricular acupuncture, acupressure was applied 8 times per day for 7 days | Vaccaria seeds | Auricular acupuncture can effectively relieve the pain of patients and is more easily accepted by patients | (Yeh et al., 2012) |
| Axial neck pain after anterior cervical discectomy and fusion | RCT | 29 | The sham-auricular point group | Shenmen (TF4),sympathetic (AH6a), subcortex (AT4), occiput (AT3), and cervical vertebra (AH13) | Each treatment was retained for 5 days, once a week for a total of 4 weeks | A 6 mm × 6 mm adhesive tape with one Semen Vaccariae | The findings supported the therapeutic effect of APA treatment on axial neck pain after ACDF surgery, and they exert the possible therapeutic effect on downregulating the levels of plasma IL-1β, IL-6, and TNF-α. | (Xia et al., 2018) |
| Chronic low back pain | Study | 61 | The sham-auricular point group | Points within the two zones for low back pain,located on the front and back of the ear, and three points known for alleviating stress and pain (shen men, sympathetic, and nervous subcortex) | Each treatment was retained for 5 days, once a week for a total of 4 weeks | Three pieces of waterproof tape (manufactured by AMIRTC) with two Vaccaria seeds (natural, nontoxic, botanical seeds with diameters of approximately 2 mm) | Auricular acupuncture can significantly improve the pain of patients with chronic low back pain | (Yeh et al., 2015) |
| Arteriovenous fistula puncture pain | RCT | 120 | Control group,compound lidocaine cream group and auricular point acupressure combined with compound lidocaine cream | Shenmen (TF4), sympathetic point (AH6a), adrenal gland (TG2), endocrine (CO18), and nervous subcortex (AT4), elbow (SF3) and wrist (SF2), heart (CO15), liver (CO12), and kidney (CO10) | Treatment was started 30 min before the start of the puncture, one ear at a time, with three days of switching, for 8 weeks | Magnetic beads patch | Auricular acupuncture can effectively reduce the pain of patients with AVF puncture, relieve their anxiety, and improve their comfort and quality of life | (Liu et al., 2021) |
| Chronic low back pain | Study | 270 | APA group (active points related to cLBP), Comparison group-1 (non-active points, unrelated to cLBP), and Comparison group-2 (enhanced educational control, an educational booklet on cLBP will be given and the treatment used by participants for their cLBP will be recorded) | Shenmen, sympathetic, and nervous subcortex | The auricular acupuncture was followed by 3 minutes of pressing each time, 3 times a day for 5 days | Vaccaria seeds | This study is expected to provide vital information on the efficacy, sustainability, and underlying mechanism of APA on cLBP necessary for APA to gain acceptance from both healthcare providers and patients, which would provide a strong impetus for including APA as part of cLBP management in clinical and home settings. | (Yeh et al., 2020) |
| Patients after pterygium surgery | Study | 60 | The sham-auricular point group | Eye and subcortex acupoints | The patients were treated 30 minutes after surgery, 4 times a day for 1 week | Auricular point sticking with Cowherb seeds (ø 1 mm) | Auricular point acupressure accelerates corneal epithelium reconstruction and suppresses postoperative pain, making it an ideal adjunct treatment for postoperative pterygium recovery. | (Cen and Yi, 2021) |
| Chronic low back pain in older adults | Study | 37 | The sham-auricular point group | Shenmen, sympathetic, and nervous subcortex | After the treatment, the acupressure was applied 3 times a day for 3 minutes each time, 5 days a week for 4 weeks | Vaccaria seeds (natural, nontoxic, botanical seeds) | Auricular point acupressure is both feasible and safe for elderly individuals with chronic low back pain to practice at home | (Yeh et al., 2014b) |
| Anal pain of mixed hemorrhoid after external excision and internal ligation | RCT | 90 | An EA group, an auricular point sticking group and a combined group | Shenmen, sympathetic, nervous subcortex and anus acupoint | Compressions were performed for 3 to 5 minutes each time, 3 to 6 times a day. The treatment lasted for 3 days | Auricular point sticking with Wangbuliuxing | Auricular acupuncture can significantly relieve pain and has a long analgesic effect | (Long et al., 2018) |
| Chronic low back pain | Study | 24 | The sham-auricular point group | Shenmen, sympathetic, nervous subcortex and corresponding acupoints (low back) | After the treatment, the acupressure was applied 3 times a day for 3 minutes each time, 5 days a week for 4 weeks | Vaccaria seeds | After auricular acupuncture, the pain intensity was reduced and the physical function was improved | (Yeh et al., 2013) |
| Acute pain | Study | 206 | The sham-auricular point group | Shenmen (TF4), Subcortex (AT4), Adrenal gland (TG2P), and two anatomical regions corresponding to the site of pain | Each acupoint was pressed for 1-2 minutes | Vaccaria seeds | Auricular acupuncture can significantly relieve the degree of acute pain in dementia patients | (Zhang et al., 2022) |
| Aromatase Inhibitor-Induced Arthralgia in Postmenopausal Breast Cancer Survivors | Study | 20 |  | Points corresponding to the body pain location  (arm, knee, or foot, depending on patient’s pain location) and three points know for alleviating stress  and pain (shenmen, sympathetic, and nervous  subcortex) | Once per week for four weeks | Vaccaria seeds | Auricular point acupressure is feasible and effective in managing arthralgia in breast cancer survivors | (Yeh et al., 2017) |
| Chronic cervical pain | Study | 19 |  | Cervical Musculature in the Posterior Wall, Thalamus, Zero, Shen Men, and Prefrontal Cortex | Auricular acupuncture was placed for 20 minutes each time, once a week for 6 weeks | Acupuncture needles 0.25 mm × 15 mm (DONGBANG, Korea) | Auricular acupuncture can be used successfully as a complementary method to treat chronic cervical pain | (Sant'Anna et al., 2021) |
| The pain after ambulatory knee surgery | RT | 120 | Invasive needle control group | Knee joint, shenmen and lung | Auricular acupuncture was retained until the following morning | Indwelling steel auricular acupuncture needles (0.22 mm diameter and 1.5 mm long) | Auricular acupuncture relieved pain and reduced the need for ibuprofen after surgery | (Usichenko et al., 2007) |
| Depression and anxiety in isolated COVID-19 patients | RCT | 68 | The sham-auricular point group | Shenmen, Subcortex, Liver and Endocrine | Participants were required to press the magnetic beads against the 4 auricular points 1 min 5 times a day for 14 days. The ear magnetic beads were changed every 3 days | Magnetic beads patch | Auricular acupressure is an effective and safe treatment for alleviating symptoms of depressive and anxiety in patients with COVID-19 | (Cai et al., 2022) |
| Patients with pain | A systematic review and meta-analysis | 806 | The sham-auricular point group |  |  |  | In these studies, auricular therapy resulted in significant pain relief compared with sham or control groups | (Yeh et al., 2014a) |
| Mild-to-moderate depression | RT | 468 | The escitalopram group | Liver and heart | Auricular acupuncture was applied once in the morning and evening every day for at least 30 minutes each time for 8 weeks | TECAS treatment (SDZ-IIB, Hwato, Suzhou Medical Appliance Factory, Suzhou, China) | Auricular acupuncture has high acceptability, better safety in improving depression and related symptoms, and is particularly effective in reducing traumatic depression. It can be used as an effective portable treatment for mild to moderate depression. | (Zhang et al., 2023) |
| Chemotherapy-induced taste alterations in patients with cancer | RCT | 49 | The acupuncture group | CO3 (Cardia), CO4 (Stom ach), CO12 (Liver), CO13 (Spleen), LO2 (Tongue), P3 (Ear dorsum spleen), AT4 (Subcortex), and appendix acupoints TF4 (Shenmen), AH6a (Sympathesis), CO17 (Tri-jiao) | After auricular acupuncture, each acupoint was pressed for 1 minute, 4 times a day for 2 weeks | Vaccaria seeds | Auricular acupuncture is a beneficial intervention for managing chemotherapy-induced taste alterations in cancer patients receiving platinum-based chemotherapy. It also helps to improve quality of life and negative mood. | (Wang et al., 2023) |
| Major depressive disorder | RT | 107 | The citalopram group | Auricular concha area | Each treatment lasted for 40 minutes, one in the morning and one in the evening, for a total of 8 weeks | An SDZ-IIB electronic stimulator (Suzhou Medical Application Factory, 12-14 West Qilin Lane, Suzhou, China) | The taVNS produced a significantly higher remission rate at week four and week six than citalopram | (Li et al., 2022) |
| Persistent Spinal Pain Syndrome (PSPS) | RCT | 51 | The placebo control group | Shenmen, subcortex, sympathetic nerves, liver, and lumbar vertebrae, | 6 weeks with 5 days of seed attachment and 2 days of rest as 1 cycle | Sinapsis alba seeds | Auricular acupressure is an effective, safe, and cost-effective non-invasive care intervention to improve sleep in patients with PSPS. | (Lim and Park, 2023) |
| College students with primary insomnia | RCT | 74 | Auricular acupoint bloodletting and auricular acupressure group | Shoulder pain / Resistance sensitive point, Pain sensitive point(areas SF1 and SF2) | Twice a week for 4 weeks | Vaccaria seed | Both auricular acupoint bloodletting + auricular acupressure and Auricular acupoint can relieve insomnia symptoms, but a stronger long-term effect were observed for auricular acupoint bloodletting + auricular acupressure. | (Chen et al., 2022) |
| Maintenance hemodialysis patients | Study | 22 |  | Shenmen (TF4), sympathetic autonomic(AH6a), heart (CO15), subcortex (AT4), and endocrine(CO18) | 5 days a week for four weeks | Vaccaria seed | Participants reported improved sleep quality, shorter sleep latency , less sleep disturbance , and less daytime dysfunction . They also exhibited less dependency on sleep medications, However, these improvements were not preserved 1 month after treatment. | (Wu et al., 2014) |
| Chronic Low Back Pain | RCT | 92 | The sham-auricular point group(stomach, mouth,duodenum, and eye acupoints of both ears). | Shenmen, sympathetic, and nervous subcortex | Each ear at least three times a day for 3 min each time,five days a week for four weeks | Vaccaria seed | Auricular point can improve the sleep quality of patients with low back pain | (Yeh et al., 2016) |
| Hemodialysis Patients with Insomnia | RCT | 133 | The sham-auricular point group | Shen Men (Spiritual Gate, TF4), Jiao Gan(Sympathetic autonomic, AH6a), Xin (Heart, CO15), Pi Zhi Xia (Subcortex,AT4), and Nei Fen Mi (Endocrine, CO18). | Three times during the day and once in the evening. The replacement of auricular pasters was performed three times a week. The treatment continued for 8 weeks. | Vaccaria seed | Auricular acupressure could serve as a complementary or alternative therapy for maintenance hemodialysis patients with insomnia by improving their sleep quality and reducing their use of hypnotics. | (Wu et al., 2022) |
| Women with postpartum insomnia | Study | 30 |  | Shenmen point | Four times a day for 14 days. | Ferrite magnets | Auricular acupressure can be an alternative complementary therapy to aid postpartum women with insomnia in improving sleep quality. | (Ko et al., 2016) |
| Female participants with perimenopausal insomnia | RCT | 70 | Auricular point pressure and fire dragon pot moxibustion group | Shenmen, subcortical, sym pathetic, endocrine, kidney, heart, liver, and spl | The participants were instructed to apply pressure 4–5 times a day, for 30–60 s per point, with more pressure before bedtime. The auricular seeds were changed once a week, alternating between the two ears, for a total of 10 weeks of treatment | Vaccaria seed | Auricular point seed burying combined with fire dragon pot moxibustion therapy can be more effect than auricular point seed burying alone in treating perimenopausal women with insomnia. | (Feng et al., 2022) |
| Insomnia | RCT | 28 | The sham-auricular point group | Shen Men, Sympathetic autonomic, Kidney, Insomnia 1,and Insomnia 2 | 5 days a week for 6 weeks | Disposable needles (0.30*13mm, VIVA®; Helio, San Jose, CA) | Auricular acupressure may have a role in the treatment of insomnia, especially in combination with other treatments such as cognitive behavioral therapy. | (Sjöling et al., 2008) |
| Elderly with insomnia | Study | 15 |  | Shenmen (or Gate of Spirit), heart, kid ney, liver, spleen, occiput, and subcortex | 3-week treatment course | Magnetic pearls | Auricular acupuncture using magnetic pearls could have a long-term effect, at least within the observed period of time, on improving the quality as well as the quantity of sleep among the elderly. | (Suen et al., 2003) |
| Breast cancer patients undergoing chemotherapy | RCT | 72 | The control group that received placebo auricular pressure on points not traditionally associated with improving sleep disturbance. | Shenmen, heart, anterior lobe, and occiput | 6 times a week for 6 weeks. | Vaccaria seed | Auricular acupuncture effectively reduced the levels of cytokines and improved sleep quality. | (Yoon and Park, 2019) |
| Patients with depression | RCT | 74 | The nonspecific auricular acupuncture group | Shenmen, subcortex, heart, lung, liver, and kidney | Both groups participated in 12 AA sessions with semipermanent needles, receiving daily stimulation twice a week for 6 weeks, and were subsequently followed up for 3 months. | The size of the semipermanent needle (Complementar) used was 0.2 mm wide and 2.5 mm long | Both groups could improve patients' anxiety state, and there was no difference in depression recovery at 3 months. However, remission rates of depression at 3 months were significantly higher in the auricular acupuncture group | (de Oliveira Rodrigues et al., 2023) |
| High-Risk Diabetes Mellitus | RCT | 69 | The control groups did not undergo auricular pellet acupressure. | Shen men, kidney point, and endocrine point. | Three times daily for 5 consecutive days. After a 2-day rest period,over a total treatment period of 20 days. | Vaccaria seeds | Auricular pellet acupressure can increase the concentration of an tioxidative enzymes in persons with high-risk DM | (Liu et al., 2008) |
| Type 2 Diabetes Mellitus | RCT | 44 | The control groups did not undergo auricular pellet acupressure. | Shenmen, Kidney, Spleen; Endocrine; Vagus Nerve; Foot, Sympathetic Nervous System, Pancreas; Liver, Hypophysis. | Five sessions that took place once a week | Semi-permanent needles from the Complementar Agulhas brand, with a 1.5 mm size | Auricular acupuncture proved to be efficient in improving circulatory conditions and plantar temperature. | (Bacelar de Assis et al., 2021) |
| Type 2 Diabetes | Study | 28 |  | Endocrine, Pancreas,Shen Men, Liver, Thalamus, Spleen (Chinese location), and Kidney | Three times per day for 12 seconds each time，received 2 weeks of treatment. | Vaccaria hispanica seeds | An auricular protocol may provide a low-cost, effective treatment for lowering blood glucose in patients with T2DM. | (Boccino, 2023) |
| Reproductive women suffer from polycystic ovary syndrome (PCOS). | RCT | 60 | The sham-auricular point group | CO10 (Shen), CO12 (Gan), CO13 (Pi), CO18 (Nei fen mi), AT4 (Pi zhixia), TF2 (Nei sheng zhi qi) and TG2 (Xia ping) | Four times per day, which are 30 min before three meals and sleep. Each time lasts at least 5 min; last for 12 weeks. | Magnetic beads (Hengshui Fengfan Medical Devices Co.) | Auricular points acupressure may serve as alternative management for PCOS for its benefits in both physical and  psychological well-being. | (Li et al., 2019) |
| Type 2 diabetes mellitus | Study | 71 |  | Cavum choncae of both ears | Each auricular point will be pressed gently 2 times lasting 1 min each time. After the whole intervention, these tapes will be removed | Ear vagus nerve stimulator (Huatuo). | The stimulation in the cavum conchae of patients with types 2 diabetes mellitus may help decrease HbA1c, BUN, SCr, TC  and AST, and may be an effective treatment for type 2 diabetes mellitus. | (Ju et al., 2014) |

References

Bacelar De Assis B, De Cássia Lopes Chaves E, De Sousa L, Machado Chianca T C, Carvalho Borges J B, Silva Vilela Terra A M, Zatiti Brasileiro T O, Mariana Fulanetti C, Fabio Cabral P, Elisama De Oliveira P, De Castro Moura C, Iunes D H (2021). The effects of auricular acupuncture on vascular parameters on the risk factors for diabetic foot: A randomized clinical trial. Complement Ther Clin Pract, 44: 101442

Boccino J (2023). Auricular Acupuncture for Lowering Blood Glucose in Type 2 Diabetes: A Pilot Study. Med Acupunct, 35(4): 186-194

Cai W, Zhang K, Wang G T, Li J, Wei X Y, Ma W, Li Y J, Wang B, Shen W D (2022). Effects and safety of auricular acupressure on depression and anxiety in isolated COVID-19 patients: A single-blind randomized controlled trial. Front Psychiatry, 13: 1041829

Cen L, Yi C (2021). Therapeutic effects of auricular point acupressure on the recovery of patients after pterygium surgery: A pilot study. Complement Ther Clin Pract, 43: 101339

Chai X M, Shi H Y, Zhang J J, Wang L, Gao H X, Dai Y L, Gao L L, Yu J Q, Li Y X, Wang C C (2022). Analgesic effect of auricular point acupressure for acute pain in patients with dementia: study protocol for a randomized controlled trial. Trials, 23(1): 404

Chen H, Zhang M J, Wu J A, She Y F, Yuan X R, Huo Y X, Sun H, Liu D N, Shi X L (2022). Effect of Auricular Acupoint Bloodletting plus Auricular Acupressure on Sleep Quality and Neuroendocrine Level in College Students with Primary Insomnia: A Randomized Controlled Trial. Chin J Integr Med, 28(12): 1096-1104

De Oliveira Rodrigues D M, Menezes P R, Machado Ribeiro Silotto A E, Heps A, Pereira Sanches N M, Schveitzer M C, Faisal-Cury A (2023). Efficacy and Safety of Auricular Acupuncture for Depression: A Randomized Clinical Trial. JAMA Netw Open, 6(11): e2345138

Feng H, Pan A, Zheng G, Yu W (2022). Clinical study of auricular point seed burying combined with fire dragon pot moxibustion in perimenopausal women with insomnia. J Obstet Gynaecol Res, 48(7): 1938-1944

Ju Y, Zhang H, Chen M, Chi X, Lan W, Zhang H, Mo J, Yung N Y (2014). Effects of auricular stimulation in the cavum conchae on glucometabolism in patients with type 2 diabetes mellitus. Complement Ther Med, 22(5): 858-863

Ko Y L, Lin S C, Lin P C (2016). Effect of auricular acupressure for postpartum insomnia: an uncontrolled clinical trial. J Clin Nurs, 25(3-4): 332-339

Li S, Rong P, Wang Y, Jin G, Hou X, Li S, Xiao X, Zhou W, Wu Y, Liu Y, Zhang Y, Zhao B, Huang Y, Cao J, Chen H, Hodges S, Vangel M, Kong J (2022). Comparative Effectiveness of Transcutaneous Auricular Vagus Nerve Stimulation vs Citalopram for Major Depressive Disorder: A Randomized Trial. Neuromodulation, 25(3): 450-460

Li Y, Du J L, Hao P L, Zhang M X, Jiang Y B, Shu M, Guan L (2021). [Effect of auricular point sticking therapy on perioperative pain in patients with partial lung resection]. Zhongguo Zhen Jiu, 41(6): 603-607

Li Y, Hou L, Wang Y, Xie L, Zhang M, Pan Z, Li Y, Ding Y, Sun M, Qu Y, Liu S (2019). Auricular points acupressure for insulin resistance in overweight/obese women with polycystic ovary syndrome: protocol for a randomised controlled pilot trial. BMJ Open, 9(5): e027498

Lim Y, Park H (2023). The Effects of Auricular Acupressure on Low Back Pain, Neuropathy and Sleep in Patients with Persistent Spinal Pain Syndrome (PSPS): A Single-Blind, Randomized Placebo-Controlled Trial. Int J Environ Res Public Health, 20(3)

Liu C F, Yu L F, Lin C H, Lin S C (2008). Effect of auricular pellet acupressure on antioxidative systems in high-risk diabetes mellitus. J Altern Complement Med, 14(3): 303-307

Liu X, Wei W, Wu Y, Jiang X, Liu X, Zhang Y, Yeh C H, Zhang Y (2021). Auricular Point Acupressure Combined with Compound Lidocaine Cream to Manage Arteriovenous Fistula Puncture Pain: A Multicenter Randomized Controlled Trial. Evid Based Complement Alternat Med, 2021: 5573567

Long Q, Li Y, Li J, Wen Y, Du W, Wan C, Yue C (2018). [Clinical observation of electroacupuncture combined with auricular point sticking therapy for anal pain of mixed hemorrhoid after external excision and internal ligation]. Zhongguo Zhen Jiu, 38(6): 580-585

Lovato A, Postiglione M, Gagliardi G, Parmagnani M, Biral M, Ceccherelli F (2023). Needle contact test in auricular acupuncture for shoulder pain and where effective auricular acupoints identified are positioned on the map: A controlled study. Eur J Transl Myol, 33(1)

Mao J J, Liou K T, Baser R E, Bao T, Panageas K S, Romero S a D, Li Q S, Gallagher R M, Kantoff P W (2021). Effectiveness of Electroacupuncture or Auricular Acupuncture vs Usual Care for Chronic Musculoskeletal Pain Among Cancer Survivors: The PEACE Randomized Clinical Trial. JAMA Oncol, 7(5): 720-727

Ruela L O, Iunes D H, Nogueira D A, Stefanello J, Gradim C V C (2018). Effectiveness of auricular acupuncture in the treatment of cancer pain: randomized clinical trial. Rev Esc Enferm USP, 52: e03402

Sant'anna M B, Sant'anna L B, Chao L W, Sant'anna F M (2021). Auriculotherapy for Chronic Cervical Pain. Med Acupunct, 33(6): 403-409

Sjöling M, Rolleri M, Englund E (2008). Auricular acupuncture versus sham acupuncture in the treatment of women who have insomnia. J Altern Complement Med, 14(1): 39-46

Suen L K, Wong T K, Leung A W, Ip W C (2003). The long-term effects of auricular therapy using magnetic pearls on elderly with insomnia. Complement Ther Med, 11(2): 85-92

Usichenko T I, Henkel B J, Klausenitz C, Hesse T, Pierdant G, Cummings M, Hahnenkamp K (2022). Effectiveness of Acupuncture for Pain Control After Cesarean Delivery: A Randomized Clinical Trial. JAMA Netw Open, 5(2): e220517

Usichenko T I, Kuchling S, Witstruck T, Pavlovic D, Zach M, Hofer A, Merk H, Lehmann C, Wendt M (2007). Auricular acupuncture for pain relief after ambulatory knee surgery: a randomized trial. Cmaj, 176(2): 179-183

Wang M, Xu Y, Wu Y, Liu C, Chen Y, Hua D, Liu Q (2023). Evaluating the efficacy of auricular acupuncture for chemotherapy-induced taste alterations: A pilot randomized controlled trial. Eur J Oncol Nurs, 67: 102458

Wu Y, Yang L, Zhong Z, Wu X, He Z, Ma H, Cai C, Li Y, Wu X, Fu B, Chen X, Wang L, Zhao D, Meng X, Qi A, Yang A, Li L, Liu X, Zou C, Lin Q (2022). Auricular Acupressure for Hemodialysis Patients with Insomnia: A Multicenter Double-Blind Randomized Sham-Controlled Trial. J Integr Complement Med, 28(4): 339-348

Wu Y, Zou C, Liu X, Wu X, Lin Q (2014). Auricular acupressure helps improve sleep quality for severe insomnia in maintenance hemodialysis patients: a pilot study. J Altern Complement Med, 20(5): 356-363

Xia B, Xie Y, Hu S, Xu T, Tong P (2018). Effect of Auricular Point Acupressure on Axial Neck Pain After Anterior Cervical Discectomy and Fusion: A Randomized Controlled Trial. Pain Med, 19(1): 193-201

Yeh C H, Chiang Y C, Hoffman S L, Liang Z, Klem M L, Tam W W, Chien L C, Suen L K (2014a). Efficacy of auricular therapy for pain management: a systematic review and meta-analysis. Evid Based Complement Alternat Med, 2014: 934670

Yeh C H, Chien L C, Balaban D, Sponberg R, Primavera J, Morone N E, Glick R, Albers K M, Cohen S M, Ren D, Huang L C, Suen L K (2013). A randomized clinical trial of auricular point acupressure for chronic low back pain: a feasibility study. Evid Based Complement Alternat Med, 2013: 196978

Yeh C H, Chien L C, Chiang Y C, Huang L C (2012). Auricular point acupressure for chronic low back pain: a feasibility study for 1-week treatment. Evid Based Complement Alternat Med, 2012: 383257

Yeh C H, Kwai-Ping Suen L, Chien L C, Margolis L, Liang Z, Glick R M, Morone N E (2015). Day-to-Day Changes of Auricular Point Acupressure to Manage Chronic Low Back Pain: A 29-day Randomized Controlled Study. Pain Med, 16(10): 1857-1869

Yeh C H, Li C, Glick R, Schlenk E A, Albers K, Suen L K, Lukkahatai N, Salen N, Pandiri S, Ma W, Perrin N, Morone N E, Christo P J (2020). A prospective randomized controlled study of auricular point acupressure to manage chronic low back pain in older adults: study protocol. Trials, 21(1): 99

Yeh C H, Lin W C, Kwai-Ping Suen L, Park N J, Wood L J, Van Londen G J, Howard Bovbjerg D (2017). Auricular Point Acupressure to Manage Aromatase Inhibitor-Induced Arthralgia in Postmenopausal Breast Cancer Survivors: A Pilot Study. Oncol Nurs Forum, 44(4): 476-487

Yeh C H, Morone N E, Chien L C, Cao Y, Lu H, Shen J, Margolis L, Bhatnagar S, Hoffman S, Liang Z, Glick R M, Suen L K (2014b). Auricular point acupressure to manage chronic low back pain in older adults: a randomized controlled pilot study. Evid Based Complement Alternat Med, 2014: 375173

Yeh C H, Suen L K, Shen J, Chien L C, Liang Z, Glick R M, Morone N E, Chasens E R (2016). Changes in Sleep With Auricular Point Acupressure for Chronic Low Back Pain. Behav Sleep Med, 14(3): 279-294

Yoon H G, Park H (2019). The effect of auricular acupressure on sleep in breast cancer patients undergoing chemotherapy: A single-blind, randomized controlled trial. Appl Nurs Res, 48: 45-51

Zhang J J, Yu L, Mei J H, Wang H X, Gao H X, Fu J F, Cheng Y, Gao L L, Bu L, Yu J Q, Wang C C, Li Y X (2022). Effect of Auricular Acupressure on Acute Pain in Nursing Home Residents with Mild Dementia: A Single-Blind, Randomized, Sham-Controlled Study. Evid Based Complement Alternat Med, 2022: 6406383

Zhang Z J, Zhang S Y, Yang X J, Qin Z S, Xu F Q, Jin G X, Hou X B, Liu Y, Cai J F, Xiao H B, Wong Y K, Zheng Y, Shi L, Zhang J N, Zhao Y Y, Xiao X, Zhang L L, Jiao Y, Wang Y, He J K, Chen G B, Rong P J (2023). Transcutaneous electrical cranial-auricular acupoint stimulation versus escitalopram for mild-to-moderate depression: An assessor-blinded, randomized, non-inferiority trial. Psychiatry Clin Neurosci, 77(3): 168-177
